# Psychometric evaluation of the Arabic version of the higher education inventory scale for nursing students

**DOI:** 10.1186/s12888-021-03082-9

**Published:** 2021-02-06

**Authors:** Dina Masha’al, Audai A. Hayajneh, Loai Issa Tawalbeh

**Affiliations:** 1 Adult Health Nursing Department, Faculty of Nursing, Jordan University of Science and Technology, P.O. Box: 3030, Irbid, 22110 Jordan; 2 Adult Health-Nursing Department, Faculty of Nursing, Jordan University of Science and Technology, P.O. Box: 3030, Irbid, 22110 Jordan; 3 Princess Salma Faculty of Nursing, Al-al-Bayt University, P.O. Box: 130049, Al-Mafraq, 25113 Jordan

**Keywords:** Psychometric, Evaluation, Arabic, Higher education inventory scale, Nursing, Students, Jordan

## Abstract

**Background:**

Studies in the literature have relied on a single instrument to assess stress levels and sources among nursing students in Jordan and in other Arab countries. Thus, there is a need to develop Arabic versions of psychometrically validated instruments for evaluating a wider range of aspects related to stress and stressors. The Higher Education Stress Inventory (HESI) is an instrument used to assess various aspects of stress and stressors related to higher education in different educational settings and among different student populations. To date, no exploratory or confirmatory factor analyses have been conducted to study the factor structure of the Arabic version of the HESI. Therefore, the current study aimed to evaluate the psychometric properties of the Arabic version of the HESI (Arabic-HESI) among nursing students in Jordan.

**Methods:**

The structure of the instrument was tested using exploratory factor analysis (EFA), confirmatory factor analysis (CFA), and maximum likelihood estimation among a sample of 355 nursing students at five Jordanian universities.

**Results:**

The Arabic-HESI proved to have excellent content validity index (CVI = 0.92). The instrument showed good internal consistency reliability (Cronbach’s α = 0.75), as well as for the two emerged factors “challenges” and “dissatisfaction” (Cronbach’s α were 0.75, 0.72 respectively). The results support the two-factor model for the Arabic-HESI, as the instrument was found to have robust structure and acceptable goodness-of-fit indices.

**Conclusion:**

The Arabic-HESI is a reliable and valid instrument for assessing stress levels and stressors among nursing students in Jordan. Using the shortened version of the HESI to assess stress among nursing students is recommended. Identifying new features of stress and stressors among nursing students in Jordan will enable universities and nursing faculties to better support their students.

## Background

Stress is a natural physical, emotional, and mental reaction to a stimulus that disturbs the normal functioning of the body [1]. It is experienced when people recognize that the demands are greater than their individual and social resources [2]. Human beings respond to stress differently depending on their social, economic, environmental, and genetic backgrounds [3].

Nursing is considered a demanding and stressful profession due to the excessive workloads, complexity of patient care activities, unorganized work environments, and lack of leader support [4]. Similarly, healthcare education, including nursing education, is stressful for students, mainly due to the vast amounts and rates of knowledge and the frequent changes in needs and services [5]. Further, healthcare education does not focus only on teaching knowledge but also on teaching skills such as problem solving, research, interpersonal interaction, psychomotor, and lifelong learning skills [5, 6]. This requires long hours of practical and theoretical study, which increases the pressure on students [5, 6]. Previous studies have indicated that nursing students have higher stress levels compared to students of other healthcare-related fields [7, 8], with education-related stress levels among nursing students ranging from medium to high [9–12]. While some stressors are considered motivational and may encourage achievement [13], experiencing prolonged stress threatens the physical, mental, and psychological health of students [6]. Furthermore, experiencing unresolved chronic stress may have adverse effects on students’ academic performance [10, 14, 15]. Eventually, high levels of unresolved stress may discourage students from pursuing nursing education, therefore impacting the nursing workforce [10].

Studies in the literature have highlighted several education-related stressors among nursing students, including academic, clinical, and personal/social stressors [9, 15, 16]. Examples of academic stressors include heavy workloads, exams and assignments, the fear of failing and achieving low grades, and the lack of sufficient guidance from tutors [11, 17–19]. Clinical stressors include students’ heavy responsibilities in clinical settings, students’ uncertainty regarding what is expected from them, the pressure of meeting the expectations of staff, the fear of making mistakes and harming patients, and criticism from peers, senior staff, and physicians [11, 17, 19]. Personal and/or social stressors entail students’ health issues, family events, lack of recreation time, high parental expectations, and financial issues [11, 17–19].

Previous studies have indicated a link between certain sociodemographic characteristics and increased stress levels among nursing students. For example, students from low-income families have been found to experience higher levels of stress in comparison to other students, as they may worry about not being able to meet their scholastic requirements, tuition fees, basic needs (i.e., food, accommodation, and transportation), or personal needs [12, 18, 20]. Further, with many universities now using blended learning and/or e-learning as a result of the COVID-19 pandemic, students from low-income families may worry about the costs of purchasing appropriate electronic devices, good internet services, and information applications [21]. Gender has also been found to impact students’ stress levels, with female nursing students frequently reporting higher levels of stress compared to male students [12, 15, 20, 22]. Previous studies have attributed this to the fact that in comparison to female students, male students are more reluctant to talk about their stress experiences, are less aware of their stress, and have less knowledge about disease detection and health promotion behaviors [23, 24]. Male students may be less able to express their feelings due to cultural norms which associate masculinity with indomitability and power [15, 24]. As for the impact of academic year on students’ stress levels, studies in the literature have reported conflicting findings. For example, Aslan and Akturk [22] and Ribeiro et al. [12] found stress levels to be higher among senior students than students in other years, mainly due to the nature of the advanced theoretical and clinical courses that senior students must take. Meanwhile, Admi and colleagues [14] found that junior nursing students experience the highest levels of stress, attributing this to their lack of knowledge and training experience required for future courses.

In Jordan, the wide spread of COVID-19, the strict national lockdown that was imposed by the government, and the transition to distance learning may have constituted new sources of stress for nursing students. In comparison to traditional education, distance learning is associated with higher stress levels among university students. The huge academic workload, the high frequency of examinations, and financial problems were sources of distance learning-related stress among university students in the study of Kwaah & Essilfie [25]. Meanwhile, Moawad [26] concluded that the main stressors affecting university students as a result of the transition to distance learning during the pandemic were uncertainty regarding exams, the semester end date, and the evaluation methods used. Further, in the study of Cao et al. [27], financial difficulties, the changes caused to daily life, and the delays in academic activities were found to increase feelings of isolation and consequently anxiety levels among university students in China [27].

Identifying stress levels, sources of stress, and the impacting factors is crucial for creating effective measures that help nursing students adapt and improve their educational performance. Moreover, identifying the stress levels and stressors experienced by students enables nursing faculties and administrators to resolve the causes of stress, support students, and gain the trust of students [19, 22]. Studies which have aimed to measure stress levels and sources among nursing students in Arab countries, including Jordan, are numerous [28–38]. However, all of these studies have used a single instrument which assesses clinical-related stress only and which was only recently psychometrically validated in the Arabic language [39]. Therefore, there is a need for a wider range of validated tools in order to ensure that various aspects of academic and clinical stress and stressors among nursing students in Arab countries are considered.

The Higher Education Stress Inventory (HESI) is a tool characterized by its ability to capture various aspects of stressors related to higher education regardless of the setting or the student population. The instrument was developed by a group of psychiatry professors in Sweden to measure stress among medical students [40]. It has previously been used to assess stress levels among medical students [40, 41] and distance learning students [42]. Although the language of instruction in all nursing schools in Jordan is English, the presence of a translated and validated instrument in Arabic (the official language of Jordan) would yield more accurate results and a better understanding of the context [39]. Considering the fact that stress levels and stressors vary depending on various sociocultural aspects [43, 44], the psychometric properties of the Arabic-HESI need to be evaluated using robust analyses like EFA and CFA. Therefore, the current study aimed to examine the psychometric properties of the Arabic-HESI, which is to be used to measure education-related stress among nursing students in Jordan, by applying EFA and CFA.

## Methods

### Participants

Three hundred and fifty-five undergraduate nursing students from five public universities located in the three regions of Jordan participated in the study. The majority of the participating students were from two universities in the North Region of Jordan (68.7%), whilst 17.2% were from two other universities in the South Region, and the remaining 14.1% were from a university located in the Central Region. Students who were enrolled full-time in nursing programs and whose first language was Arabic were recruited, whilst students who had dropped out for the semester during which data were collected and students whose first language was not Arabic were excluded from the study. The mean age of the students was 20.02 years (SD = 1.12), 72.7% of the students were female, and most of the students were in their second year of study (62.54%). The sociodemographic characteristics of the students are displayed in Table 1.

**Table 1:** Nursing students’ sociodemographic characteristics

Students’ characteristics	*N*	*%*
Age (*M* = 20.02, *SD* = 1.12)		
Gender		
Male	97	27.32
Female	258	72.68
Family Monthly Income		
Very low income	222	62.54
Low income	115	32.39
Medium to high income	18	5.07
Academic year		
First year	60	16.90
Second year	222	62.54
Third year	41	11.55
Fourth year	32	9.01
Number of courses enrolled in		
Four courses	55	15.49
Five courses	169	47.61
Six courses	90	25.35
Seven courses	41	11.55
Clinical courses/Lab		
Yes	338	95.21
No	17	4.89
Type of electronic device		
Smart phone	300	84.51
Other (Desktop Computer, laptop, tablet)	55	15.49
Internet service type		
Prepaid package	215	60.56
Home internet with limited download	82	23.10
Others (Home internet with unlimited download, Fiber,….)	58	16.34
Is purchasing internet services a financial burden?		
Yes	299	84.23
No	56	15.77
Do you have your own space to study at home?		
Yes	142	40
No	213	60

*M* Mean, *SD* Standard deviation

### Procedure

Google forms was used to create an online survey. After institutional review board approval from the authors’ university was obtained and the facilitating requests from the other universities were granted, the survey was distributed to nursing students through the websites of the five universities. The participants were informed that their participation was voluntary and that they had the right to withdraw from the study at any time without consequences. Further, the contact details of the researchers were provided in case the participants had any questions. The survey included the HESI items in Arabic and questions about the participants’ sociodemographic characteristics. Students who agreed to participate in the study were asked to complete the survey and click the “submit” button at the end.

### Measures

The HESI was developed by Dahlin et al. [40] to measure higher education related stress among medical students. The inventory is neutral to educational settings, which allows for the comparison between different student populations. Four of the inventory items were borrowed and three of them were slightly modified from the Perceived Medical School Stress (PMSS) scale [45]. The inventory consists of 33 items which describe the presence and absence of stressful aspects in higher education. The items are scored on a 4-point Likert scale, where 1 = totally disagree, 2 = somewhat disagree, 3 = somewhat agree, and 4 = totally agree. The items 2, 6, 8, 10, 13, 17, 19, 26, 27, and 33 are reversed items because they indicate the absence of stress. The original HESI factor analysis identified seven factors and included 24 items. The total variance explained by the factors was 48.7%. The Cronbach’s α was .85 for the instrument and was satisfactory for the seven factors as follow: worries about future endurance/competence (α = .78); nonsuppurative climate (α = .71); faculty shortcomings (α = .69); workload (α = .65); insufficient feedback (α = .65); low commitment (α = .62); and financial concerns (α = .59).

Further, students were asked to answer an open-ended question about the sources of stress they had experienced as a result of the transition to distance learning during the COVID-19 pandemic. Questions regarding the students’ sociodemographic characteristics and the circumstances of distance learning were also included in the survey.

### Translation

After permission for use and translation was granted from the original author, the English version of the HESI was translated into Arabic by two independent bilingual nursing professors. One of the professors is specialized in psychiatry and the other in education, and both professors are fluent in both Arabic and English. Then, the HESI was back-translated into English by another professor specialized in translation. The two English versions of the HESI were then compared by a native speaker, showing no differences. The translated HESI was evaluated by 4 nursing experts who have experience in tools translation and validation. The researchers were asked to rate each instrument item on a 4-point Likert scale ranging from 1 = “not relevant” to 4 = “highly relevant”. The content validity index (CVI) was 0.92. The translated HESI was piloted on 15 nursing students from one of the five universities, and no significant issues were reported by the students.

### Statistical analysis

Statistical analysis was performed using the Statistical Package for the Social Sciences (SPSS) version 25 (SPSS, Inc., Chicago, Ill) and Amos (Version 23.0) [Computer Program]. Chicago: IBM SPSS. The assumptions of normality, linearity, homogeneity, and homoscedasticity were checked for any violations. Reliability and validity analyses of the Arabic-HESI were performed by measuring the internal consistency reliability analysis (Cronbach’s α), exploratory factor analysis (EFA), and confirmatory factor analysis (CFA). EFA is the first step in validating a translated instrument that is to be used for the first time on a new population (i.e., Jordanian nursing students in the case of the current study) [46]. EFA was used to explore the underlying factor structure of the Arabic-HESI in order to examine the ability of the individual items to reflect stress. Preacher and MacCallum’s guidelines [47] were used to conduct the EFA analyses. The CFA was performed using maximum likelihood estimation to confirm the EFA structure and investigate the goodness-of-fit indices of the yielded model. In order to examine the model’s goodness-of-fit, a number of statistics were used: overall χ2, root mean square error of approximation (RMSEA) [48], comparative fit index (CFI), and the Tucker-Lewis index (TLI) [49]. CFA provides a theory-driven approach for establishing construct validity by assigning items to their corresponding factors based on theoretical beliefs [50].

The open-ended question was analyzed using content analysis [51]. The students’ responses were reviewed separately by three researchers who are experts in qualitative research. The researchers then worked in collaboration to code and categorize the responses into themes. Descriptive analyses were used to describe the sample.

## Results

### Reliability

The HESI has never been translated into Arabic, nor has it been validated among Arab populations such as the Jordanian population. Therefore, the translated version needs to establish its psychometric properties in that new population [46]. Cronbach’s alpha was used to examine the internal consistency of the Arabic-HESI. Any value above 0.7 is usually considered an acceptable reliability value for any given scale [52]. The Cronbach’s alpha of the total score of the scale, which comprises 33 items, was 0.767. However, the inter-item correlation of these items was very low (0.095) (Table 2), raising questions regarding the dimensionality of the scale with these 33 items. As a result, exploratory factor analysis (EFA) was used to determine the number of underlying factors that these 33 items are loading on, and if some of these items are not loading adequately to be well-correlated with their factors or subscales. The EFA is discussed in a separate section below. Iterated EFA results suggested the use of a two-factor model to reach the simple structure of the scale.

**Table 2:** Scale reliability statistics

	Mean	SD	Cronbach’s α	Inter-Item correlation
Scale	2.568	0.590	0.767	0.095

Of the observations, 355 were used, 0 were excluded listwise, and 355 were provided

**Fig. 1:**
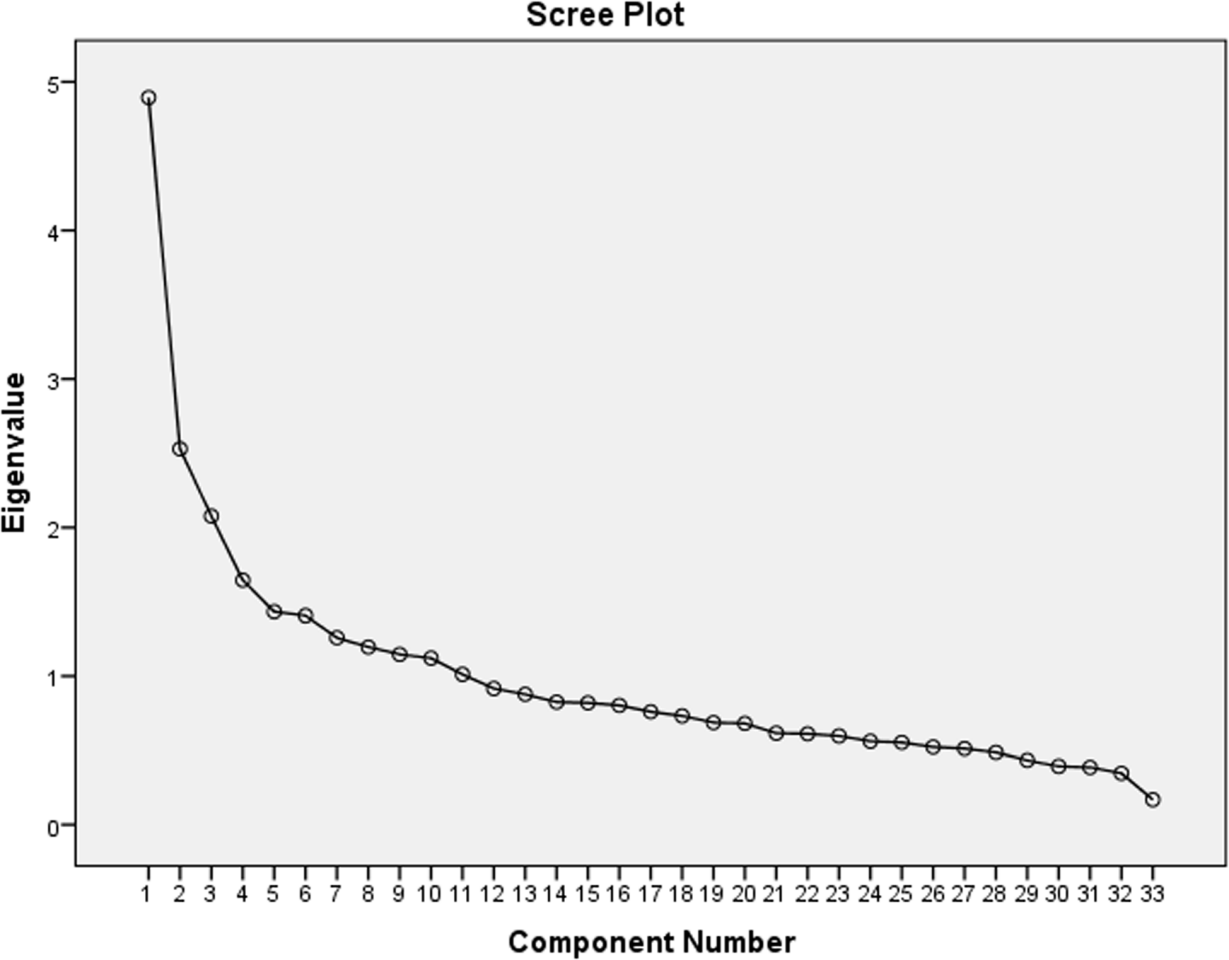
Scree plot

The EFA was used to explore the underlying factor structure of the original scale of the HESI based on Preacher and MacCallum’s [47] guidelines. A parallel analysis suggested 9 factors, and the Kaiser method and scree plot suggested three factors (Fig. 1). Lastly, the dimensionality of the underlying theoretical framework of the original scale has never been demonstrated in the literature. Therefore, the EFA of the Arabic-HESI was carried out using three factors, as suggested by the Kaiser method and scree plot. A maximum likelihood examination with a Promax rotation was used due to the probability of a correlation between the factors, with 0.30 set as the cut-off point for factor loading [53]. Thirty-three items were tested using the EFA analysis, and the item loadings were yielded, as shown in Table 3.

**Table 3:** Exploratory factor analysis of the Arabic version of HESI, three-factor model, 33 items (*N* = 355). Thirty-three items loadings of the Arabic version of HESI

	Factor 1	Factor 2	Factor 3	Uniqueness
Student union activities promote a sense of community and contribute to a better working environment for students	.	0.464	.	0.763
Studies control my life and I have little time for other activities	.	.	.	0.958
The pace of studies is too high	.	.	.	0.956
I am worried about accommodation	0.460	.	.	0.777
There is a competitive attitude among students	.	0.537	.	0.701
The studies have created anonymity and isolation among students	.	.	.	0.827
I feel that the studies have played a role in creating a cold and impersonal attitude among students	.	.	.	0.895
I meet many future colleagues that seem dejected or dissatisfied in their profession	0.335	.	.	0.849
The teachers give encouragement and personal attention	.	0.517	.	0.667
I feel that I am less well treated because of my ethnic background	0.365	.	.	0.829
The teachers often fail to clarify the aims of the studies	0.380	0.400	.	0.612
The teachers often give feedback on the students’ knowledge and skills	.	0.415	.	0.797
My fellow students support me	.	0.367	.	0.836
As a student, my financial situation is a worry	0.431	.	.	0.784
I am worried about my future economy and my ability to repay student loans	0.499	.	.	0.742
I am able to influence the studies	.	.	0.313	0.824
I feel that I am less well treated because of my sex	.	.	.	0.926
The literature is too difficult and extensive	0.424	.	.	0.796
I worry about long working hours and responsibilities in my future career	0.349	.	.	0.797
I am satisfied with my choice of career	0.327	.	.	0.810
I am worried that I will not acquire all the knowledge needed for my future profession	0.422	.	.	0.750
The studies stimulate my personal development	.	0.426	.	0.810
The professional role presented in the training conflicts with my personal views	.	0.366	.	0.764
I am proud of my future profession	.	.	0.787	0.348
As a student you are often expected to participate in situations where your role and function is unclear	0.355	.	.	0.829
I am satisfied with my choice of career	.	.	0.825	0.311
There is too much focus on passive learning of facts and too little on active seeking of knowledge and time for reflection	0.357	.	.	0.836
I feel that my teachers treat me with respect	.	0.416	.	0.699
The training demands that I join in situations that I find unethical	0.339	.	.	0.882
I feel that the training is preparing me well for my future profession	.	.	0.313	0.813
The training is characterized by an atmosphere where weakness and personal shortcomings are not accepted	.	.	.	0.914
The education is highly characterized by group activities with unclear goals and with too much responsibility placed on the student	0.453	.	.	0.813
The insight I have had into my future profession has made me worried about the stressful workload	0.507	.	0.326	0.640

As shown in Table 3, six items did not adequately load above 0.30. These items were: “My studies control my life and I have little time for other activities”, “The pace of the studies is too fast”, “The studies have created anonymity and isolation among students”, “I feel that the studies have played a role in creating a cold and impersonal attitude among students”, “I feel that I am less well treated because of my sex”, and “The training is characterized by an atmosphere where weakness and personal shortcomings are not accepted”. Thus, these items were removed before re-running the EFA analysis. Then, the number of factors was reduced to two in order to achieve the simple structure of the EFA, which necessitates at least three items loading on the same factors. Then, after the EFA was repeated for the remaining items, 7 items did not load above 0.30. These items were: “I meet many future colleagues that seem dejected or dissatisfied with their profession”, “The teachers often give feedback on the students’ knowledge and skills”, “My fellow students support me”, “I worry about the long working hours and responsibilities of my future career”, “The studies stimulate my personal development”, “The training demands that I join in situations that I find unethical”, and “The insight I have gained into my future profession has made me worried about the stressful workload”. These items were removed before re-running the EFA analysis, after which one item (“Student union activities promote a sense of community and contribute to a better working environment for students”) did not load above 0.30. This item was then removed before re-running the EFA analysis for the remaining items. Lastly, the EFA simple structure of the two-factor model was achieved with 18 items, as shown in Table 3. Factor one had 14 items and was named “challenges”, and factor two had 4 items and was named “dissatisfaction”.

The two reliability values of factors one and two were 0.758 and 0.717, respectively; as shown in Table 4, these values are considered good. After the removal of 17 items, as suggested by the iterated EFA, the final Arabic version of the HESI comprised 16 items (Table 5). The total scale reliability of the Arabic version of the HESI was 0.75, which is considered good.

**Table 4:** The two factors model of the Arabic version of HESI

	Challenges	Dissatisfaction	Uniqueness
I am worried about accommodation	0.484	.	0.783
There is a competitive attitude among students	0.377	.	0.851
The teachers give encouragement and personal attention	0.416	.	0.755
I feel that I am less well treated because of my ethnic background	0.411	.	0.841
The teachers often fail to clarify the aims of the studies	0.574	.	0.654
As a student, my financial situation is a worry	0.503	.	0.764
I am worried about my future economy and my ability to repay student loans	0.530	.	0.738
I am able to influence the studies	.	0.358	0.849
The literature is too difficult and extensive	0.397	.	0.830
I am worried that I will not acquire all the knowledge needed for my future profession	0.488	.	0.780
The professional role presented in the training conflicts with my personal views	0.446	.	0.790
I am proud of my future profession	.	0.940	0.184
As a student you are often expected to participate in situations where your role and function is unclear	0.370	.	0.856
I am satisfied with my choice of career	.	0.859	0.320
There is too much focus on passive learning of facts and too little on active seeking of knowledge and time for reflection	0.315	.	0.869
I feel that my teachers treat me with respect	0.460	.	0.724
I feel that the training is preparing me well for my future profession	.	0.301	0.904
The education is highly characterized by group activities with unclear goals and with too much responsibility placed on the student	0.337	.	0.895

**Table 5:** Two factors model: item reliability statistics

	M	SD	Item-total correlation	If item dropped
				Cronbach’s α
**Factor 1: Challenges**				
C1: I am worried about accommodation	2.172	1.128	0.399	0.654
Removed: There is a competitive attitude among students	2.687	1.031	0.324	0.758
C2: The teachers give encouragement and personal attention	2.546	0.911	0.319	0.667
C3: I feel that I am less well treated because of my ethnic background	1.465	0.807	0.339	0.665
C4: The teachers often fail to clarify the aims of the studies	3.028	0.809	0.452	0.651
C5: As a student, my financial situation is a worry	3.127	0.929	0.412	0.654
C6: I am worried about my future economy and my ability to repay student loans	2.501	1.103	0.442	0.647
C7: The literature is too difficult and extensive	3.420	0.726	0.361	0.664
C8: I am worried that I will not acquire all the knowledge needed for my future profession	3.569	0.699	0.373	0.663
C9: The professional role presented in the training conflicts with my personal views	2.701	0.924	0.306	0.669
C10: As a student you are often expected to participate in situations where your role and function is unclear	2.932	0.729	0.323	0.668
C11: There is too much focus on passive learning of facts and too little on active seeking of knowledge and time for reflection	3.093	0.795	0.330	0.667
C12: I feel that my teachers treat me with respect	1.775	0.802	0.423	0.655
C13: The education is highly characterized by group activities with unclear goals and with too much responsibility placed on the student	2.969	0.878	0.304	0.670
Factor 1 (**Cronbach’s a after removal = 0.758**)	2.713	0.593		
**Factor 2: Dissatisfaction**				
S1: I am able to influence the studies	1.901	0.826	0.314	0.673
S2: I am proud of my future profession	1.420	0.760	0.651	0.454
S3: I am satisfied with my choice of career	1.625	0.869	0.601	0.470
Removed: I feel that the training is preparing me well for my future profession	1.828	0.864	0.252	0.717
Factor 2 (**Cronbach’s a after removal = 0.717**)	1.694	0.217		
**Total scale (Cronbach’s a after removal = 0.75)**				

However, Table 5 of the item reliability statistics suggested that removing each of the items “There is a competitive attitude among students” and “I feel that the training is preparing me well for my future profession” would increase the Cronbach’s alpha values of their subscales and factors from 0.686 to 0.758, and from 0.659 to 0.717, respectively. The two items were therefore removed, resulting in 13 items under factor one and 3 items under factor two. The total scale Cronbach’s alpha value of the two-factor model of the Arabic version of the HESI was 0.75 (Table 5).

**Fig. 2:**
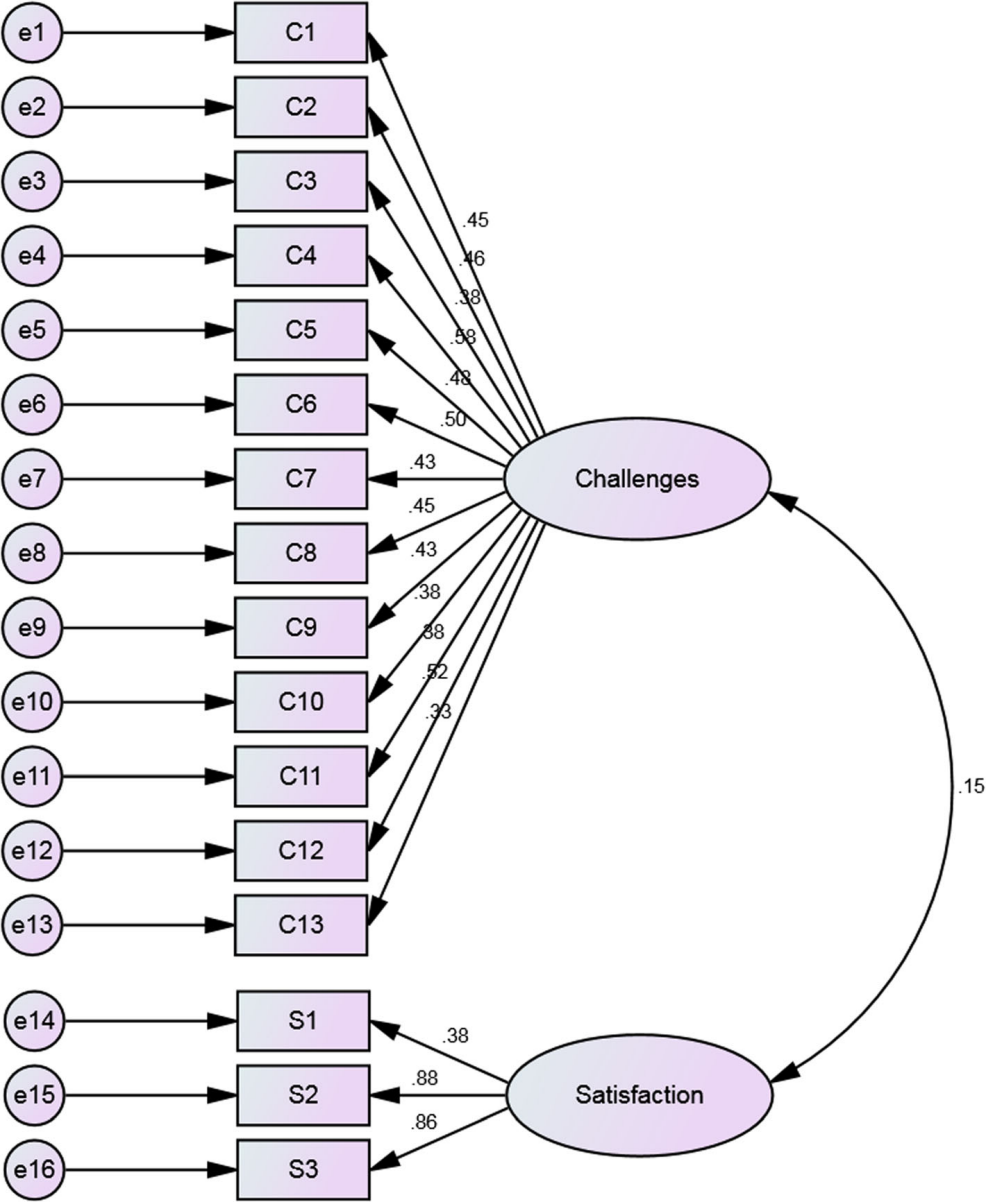
The confirmatory factor analysis of the two factors model

Confirmatory factor analysis (CFA) was conducted using the AMOS software based on a two-factor model, as suggested by the EFA (Fig. 2). All factor loadings of the latent variables displayed in Fig. 2 were above 0.3. In comparison to the three-factor model displayed in Table 3, the confirmatory factor analysis of the two-factor model showed a good fit, with the RMSEA (0.068) ranging between 0.058 and 0.078. Also, the CFI and TLI were 0.841 and 0.803, respectively, indicating an acceptable model and accounting for 45% of the total variance (Table 6). CFA incorporates the specification of one or more hypothesized models and each one proposes a group of factors (latent variables) to explain for covariance among a number of observed indicators. The latent factors are enclosed in the circle (dissatisfaction and challenges), the observed indicators (items) are enclosed in the rectangle, and the measurement error is enclosed in ellipses. Referring to Fig. 2 and Table 6, the model is determined by two interrelated constructs, challenges and dissatisfaction, which are connected with a double headed arrow indicating the correlation (*r* = 0.15). The one-headed arrow passing from the factor to the item is the regression path, which is the link between the factor and its related items. This coefficient is the factor loading. The one-headed arrow from the ellipses to the rectangle is the measurement error.

**Table 6:** Goodness-of-fit statistics for two-factors model vs. three-factors model

Fit Statistics	Two-factors model	Three-factors model
RMSEA (CI 90%)	0.068 (0.058, 0.078)	0.068 (0.062–0.74)
TLI	0.803	0.698
CFI	0.841	0.726

*RMSEA* Root mean square error, *CFI* Comparative fit index, *TLI* Tucker-Lewis index, *CI* Confidence interval

The open-ended question revealed four themes related to the sources of stress among nursing students in relation to the transition to distance learning during the COVID-19 pandemic. The first theme was high unorganized workload, which encompassed complaints about the huge daily workload, the compressed delivery of the curriculum over a short period of time, and the overlap in online meeting times. The second theme, the lack of a uniformed distance learning strategy, included student complaints about the use of multiple learning methods and applications that were new to students and teachers, the use of unclear assessment methods, and the online delivery of clinical courses. The third identified theme was limited resources, including the limited financial and physical resources of the students. This theme also included the limitations in the technical infrastructure needed for the success of distance learning, whereby students complained about poor internet services and learning management systems. Distracting environment, which was the fourth and final theme, was related to the fear of COVID-19, the contradictory COVID-19-related news, and the noisy home environment.

## Discussion

The stress experienced by nursing students in Jordan and in other Arab countries has been examined in many studies. However, to our knowledge, all studies that have been conducted in the Arab world have used a single instrument evaluating only clinical-related stress among nursing students. Although English is the language of instruction in Jordan, there are limitations in using non-Arabic psychometrically evaluated instruments, due to language and cultural variations among students. Therefore, this study aimed to assess the psychometric properties of the Arabic version of the HESI. The translation of the HESI from English into Arabic has proved successful. This result is supported by the excellent CVI and the panel of experts’ agreement that the items of the Arabic-HESI adequately measured the sources of stress among nursing students [54].

The EFA yielded a two-factor model with 16 items reflecting different stressors perceived by nursing students in Jordan. A total of 45% of the variance was explained by the model, compared to the 24-item and 22-item seven-factor models of the original and Korean versions, which explained 48.7 and 45.8% of the variance, respectively. The original HESI and the Korean HESI yielded seven factors, with variations in the items under the factors and in the significance of the factors to the construct of higher education [40, 44]. As for the Arabic-HESI, the items were grouped under two factors, namely “challenges and “dissatisfaction”. The overall internal consistency of the Arabic-HESI was 0.75, compared to 0.85 for the original HESI and 0.78 for the Korean HESI. The Cronbach’s alpha values for the subscales of the Arabic-HESI were higher in comparison to the factors of the original HESI, whilst they were close to the Cronbach’s alpha values of most of the factors of the Korean HESI [40, 44]. This may be related to the variations in the number of items under each factor in the three versions.

According to the Cronbach’s alpha results, the 13-item “challenges” factor was stronger than the 3-item “dissatisfaction” factor. The “challenges” factor items were grouped from 6 out of 7 factors of the original HESI, in addition to two other factors that were not loaded in any of the 7 factors. This may be an indication of the comprehensiveness of the “challenges” factor. Although the “financial concerns” factor in the original HESI was the weakest, the items of this factor were grouped under the “challenges” factor in the Arabic-HESI. These items were more important in the Arabic-HESI due to the financial hardship experienced by many families in Jordan, as Jordan is a developing country [18, 55, 56]. In Jordan, about 84.9% of the population live below the poverty line [56]. Further, the indications of financial concerns for the participating students were clear. For example, about 95% of our sample came from low- and very low-income families, and more than 84% claimed that purchasing internet services in order to keep up with the requirements of distance learning constituted an additional financial burden. Also, the limited financial and physical resources of the students were indicated by the ‘limited resources’ theme that was identified from the students’ responses to the open-ended question.

The “dissatisfaction” factor included the items of the “low commitment” factor from the original HESI and 50% of the items of the “low commitment” factor from the Korean HESI. The presence of these items emphasizes the importance of improving the educational environment in order to improve the experience and satisfaction levels of students, therefore reducing students’ stress levels [14]. Aslan & Akturk [22] and Hamaideh et al. [34] found that nursing students who choose nursing willingly and/or come to like it during their time in education experience lower stress levels than students who do not. Further, the items under the “dissatisfaction” factor highlighted the stereotypes about nursing held by some Jordanians. Despite the evolution of Jordanian society’s view of nursing and nursing education over the past 70 years, some people in Jordan still do not consider nursing to be a prestigious major [57]. To the best of our knowledge, many students in Jordan do not choose to study nursing willingly; rather, for many students, nursing is assigned to them by the unified admissions program based on their grades in the General Secondary Education Certification Examination (Tawjihi) [58, 59].

Items which present peer relationships as being a stressor, such as “The studies have created anonymity and isolation among students”, “I feel that the studies have played a role in creating a cold and impersonal attitude among students”, “My fellow students support me” (reversed item), and “There is a competitive attitude among students”, were removed during analysis. Social support from peers has been found to decrease students’ perceived stress during education [60], and in the context of Jordanian culture, relationships between students are characterized by strong and positive bonds [39]. Furthermore, items related to clinical training stressors, such as “The training is characterized by an atmosphere where weakness and personal shortcomings are not accepted”, “The training demands that I join in situations that I find unethical”, and “I feel that the training is preparing me well for my future profession”, were also removed. This may be attributed to the fact that data collection took place during the period of transition to distance learning due to the COVID-19 pandemic, whereby practicum courses were taught that semester (second semester 2019/2020) using videos, computer simulations, and online quizzes and assignments. Students did not have the chance to practice in clinical settings and therefore did not experience the stressors related to actual training in clinical settings. However, the qualitative results revealed that nursing students had concerns regarding the termination of clinical training in actual clinical settings. Students were dissatisfied with the way that clinical courses were being delivered and felt that they were losing out on a golden opportunity to meet real patients and acquire psychomotor skills in real clinical settings.

Furthermore, the lack of training in clinical settings may have also reduced the students’ perceived levels of stress regarding the stressors and responsibilities of their future profession. Therefore, the items “I meet many future colleagues who seem dejected or dissatisfied with their profession”, “I worry about the long working hours and responsibilities of my future career”, and “The insight I have gained into my future profession has made me worried about the stressful workload” were removed during analysis.

### Limitations

This study is not without limitations. The cross-sectional design of the study did not provide information about causal relationships among the variables. Also, the fact that this was a secondary data analysis hindered the researchers from examining the test-retest reliability, stability reliability, criterion-related validity, known-group validity, and convergent validity. Further, data were collected soon after the sudden and dramatic transition by all universities in Jordan to distance learning due to the COVID-19 pandemic. Despite the HESI being neutral to educational settings and student populations, we believe that the removal of many of the items in the Arabic-HESI during analysis may have been due to this sudden transition. Therefore, using the Arabic-HESI in normal circumstances to verify the impact of the removal or re-development of the low-loading items on the complete HESI performance is recommended.

## Conclusion

This study has provided initial evidence for the validity and reliability of the Arabic-HESI. The instrument showed good internal consistency reliability and excellent content validity. Using the reduced version of the HESI to assess stress among nursing students is recommended. In order to boost the results of the exploratory analysis of this study, future studies should use the Arabic-HESI on a larger sample size, during regular education, and using advanced confirmatory analysis. Identifying different aspects of stress and stressors among nursing students using a new instrument will enrich our knowledge and enable universities and nursing faculties in Jordan and the Arab world to use different strategies to create a new support system for students. These strategies will focus on the main sources of stress among this cohort of students and will be in line with Arab culture.

## Data Availability

The raw data can be requested from the first author: Dina Masha’al, PhD, MSN, RN.

## Abbreviation

HESI: Higher Education Inventory Scale
